# Docking-Based Virtual Screening Enables Prioritizing Protein Kinase Inhibitors With *In Vitro* Phenotypic Activity Against Schistosoma mansoni

**DOI:** 10.3389/fcimb.2022.913301

**Published:** 2022-07-05

**Authors:** Bernardo Pereira Moreira, Izabella Cristina Andrade Batista, Naiara Clemente Tavares, Tom Armstrong, Sandra Grossi Gava, Gabriella Parreiras Torres, Marina Moraes Mourão, Franco H. Falcone

**Affiliations:** ^1^ Institut für Parasitologie, Biomedizinisches Forschungszentrum Seltersberg (BFS), Justus-Liebig-Universität Giessen, Giessen, Germany; ^2^ Grupo de Helmintologia e Malacologia Médica, Instituto René Rachou, Fundação Oswaldo Cruz-FIOCRUZ, Belo Horizonte, Brazil; ^3^ School of Chemistry, University of Nottingham, Nottingham, United Kingdom

**Keywords:** *Schistosoma mansoni (S. mansoni)*, protein kinases, kinase inhibitor, *in silico* drug screening, *in vitro* phenotypic screening, ATP pocket, molecular docking

## Abstract

Schistosomiasis is a parasitic neglected disease with praziquantel (PZQ) utilized as the main drug for treatment, despite its low effectiveness against early stages of the worm. To aid in the search for new drugs to tackle schistosomiasis, computer-aided drug design has been proved a helpful tool to enhance the search and initial identification of schistosomicidal compounds, allowing fast and cost-efficient progress in drug discovery. The combination of high-throughput *in silico* data followed by *in vitro* phenotypic screening assays allows the assessment of a vast library of compounds with the potential to inhibit a single or even several biological targets in a more time- and cost-saving manner. Here, we describe the molecular docking for *in silico* screening of predicted homology models of five protein kinases (JNK, p38, ERK1, ERK2, and FES) of *Schistosoma mansoni* against approximately 85,000 molecules from the Managed Chemical Compounds Collection (MCCC) of the University of Nottingham (UK). We selected 169 molecules predicted to bind to SmERK1, SmERK2, SmFES, SmJNK, and/or Smp38 for *in vitro* screening assays using schistosomula and adult worms. In total, 89 (52.6%) molecules were considered active in at least one of the assays. This approach shows a much higher efficiency when compared to using only traditional high-throughput *in vitro* screening assays, where initial positive hits are retrieved from testing thousands of molecules. Additionally, when we focused on compound promiscuity over selectivity, we were able to efficiently detect active compounds that are predicted to target all kinases at the same time. This approach reinforces the concept of polypharmacology aiming for “one drug-multiple targets”. Moreover, at least 17 active compounds presented satisfactory drug-like properties score when compared to PZQ, which allows for optimization before further *in vivo* screening assays. In conclusion, our data support the use of computer-aided drug design methodologies in conjunction with high-throughput screening approach.

## Introduction

Schistosomiasis is a neglected tropical disease caused by trematodes of the genus *Schistosoma*. Its transmission is reported in 78 countries, and it is estimated that 700 million people who live in endemic areas are at risk of being infected (World Health Organization, 2020). This disease causes anemia, impaired childhood development, altered cognitive functions, and can induce disabling systemic morbidity, causing Disability Adjusted to Life Year (DALY) that varies between 1.7 to 4.5 million (Boros, 1989; Steinmann et al., 2006).

For almost 50 years, the treatment and control of schistosomiasis have relied on praziquantel (PZQ), a drug that has been heavily used in mass drug administration programs as well as for preventive chemotherapy strategies in highly endemic areas since 2006 (Vale et al., 2017). This drug has a low cost, few adverse effects, and it is administered in a single dose to treat all *Schistosoma* species (Gönnert and Andrews, 1977; Doenhoff et al., 2008; Vale et al., 2017). However, the effectiveness of PZQ is dependent on the mating state, sex of adult worms, and the host immune system. Furthermore, eggs and immature worms are insensitive to PZQ, thus the effectiveness of this drug also depends on the development time of infection (Pica-Mattoccia and Cioli, 2004; Doenhoff et al., 2008). In addition to these limitations, previous studies showed that *Schistosoma mansoni* may present decreased sensitivity to PZQ after exposure to multiple doses of the drug, and this reduced sensitivity can be observed in the following generations (Fallon and Doenhoff, 1994; Ismail et al., 1996; Melman et al., 2009).

In light of this situation, a search for new alternatives for schistosomiasis treatment is necessary. The latest developments in genomics, transcriptomics, and proteomics in the schistosomiasis field have boosted the search for vaccine candidates, although no successful and definitive alternative has yet been developed for this disease (Berriman et al., 2009; Han et al., 2009; Protasio et al., 2012; Tebeje et al., 2016; Sotillo et al., 2017). The discovery and development of a new drug are costly and demand several years of research. Nevertheless, the growth in computational power has facilitated an expansion in the capabilities and applicability of *in silico* studies to the drug discovery process. Among the computational tools available for the identification of candidate molecules, molecular docking is successfully used to predict ligand-target interactions at a molecular level, their binding affinities, and the orientations of the docked ligands at the active site of the target protein (Pagadala et al., 2017; Pinzi and Rastelli, 2019). Thus, the combination of *in silico* data output followed by *in vitro* testing allows the prioritization of compounds from large libraries with the potential to inhibit a single or even multiple biological targets.

Considering the need for alternative drugs against schistosomiasis, we sought to perform *in silico* screening followed by *in vitro* assays with live worms against a set of compounds predicted to target *S. mansoni* kinases. Protein kinases (PKs) are well-characterized cell signaling molecules. They play an essential role in cellular activation processes by phosphorylation of serine, threonine, and/or tyrosine residues with ATP as a source of phosphate, thus activating diverse proteins (Chang and Karin, 2001; Manning et al., 2002). Phosphorylation events are vital molecular processes for blood fluke biology and changes in phosphorylation levels can critically affect protein functions, thus reducing the survivability of parasites (Chienwichai et al., 2020). Previous studies with *S. mansoni* showed that around 2% of the predicted proteome correspond to PKs (Andrade et al., 2011; Grevelding et al., 2018), and some of them are described as being essential for the parasite’s biology (Beckmann and Grevelding, 2010; Long et al., 2010; Andrade et al., 2014; Zhao et al., 2017; Avelar et al., 2019; Tavares et al., 2020; Wang et al., 2020).

Additionally, important roles have been attributed to several Mitogen-Activated Protein kinases (MAPKs) in *S. mansoni*. For instance, c-Jun N-terminal kinase (SmJNK) is a key regulator of parasite maturation and survival. The knockdown of SmJNK in schistosomula significantly increased the parasite’s mortality, and the recovered adult worms showed considerable morphological alterations, especially in the tegument (Andrade et al., 2014). When the same strategy was used for the knockdown of extracellular signal-regulated kinases (SmERK1 and 2) low egg numbers were recovered from infected animals, and adult female worms presented severe alterations in the reproductive system (Andrade et al., 2014). More recently, Smp38 knockdown in schistosomula by RNAi affected parasite survival and development, leading to lower egg output, damaged tegument in adult worms, and undeveloped female ovaries (Avelar et al., 2019). Additionally, transcriptional analysis after Smp38 knockdown revealed that this kinase is important for the regulation of key signaling processes in the parasite, such as homeostasis and antioxidant defenses (Avelar et al., 2019). In addition, SmJNK and Smp38 pathways regulate shared genes and therefore, a simultaneous inhibition could improve inhibitory effects (Avelar et al., 2019). Moreover, the Tyrosine Kinase (TK) feline sarcoma (SmFES) is another PK validated as a potential drug target to support schistosomiasis treatment. After knockdown by RNAi, SmFES was demonstrated to participate in *S. mansoni* survival and reproduction, such as in adult worm pairing and subsequent female worm maturation. Besides, SmFES knockdown caused a significant reduction in granuloma formation (Tavares et al., 2020).

Due to the importance of protein kinases in the biology of *Schistosoma*, the present study aimed at searching for molecules that target *S. mansoni* kinases and can be used as a starting point for the development of new drugs for schistosomiasis treatment. Molecular docking was employed to identify compounds that are predicted to bind to the ATP binding site of SmERK1, SmERK2, Smp38, SmJNK, and/or SmFES. Subsequently, the selected compounds were used in *in vitro* screening assays against schistosomula and adult worms of *S. mansoni*.

## Materials and Methods

### Parasites

*S. mansoni* cercariae from the LE strain were obtained from the Mollusk rearing facilities “Lobato Paraense” of René Rachou Institute, FIOCRUZ. Cercariae were mechanically transformed into schistosomula according to Milligan and Jolly (2011) with modifications. After transformation, 100 parasites were maintained in 96-well plates with 200 µL of Glasgow Minimum Essential Medium (GMEM) (Merck) supplemented with 0.1% glucose (Vetec), 0.1% lactalbumin (Merck), 20 mM of HEPES (Merck), 2% inactivated fetal bovine serum (Gibco, ThermoFisher Scientific), 0.5% MEM vitamin solution (Gibco, ThermoFisher Scientific), 5% Schneider’s Insect Medium (Merck), 0.2 mM of triiodothyronine (Merck), 0.5 mM of hypoxanthine (Merck), 1 mM of hydrocortisone (Merck), and 1% penicillin/streptomycin (Gibco, ThermoFisher Scientific). Schistosomula culture was incubated for 24 hours in a biological oxygen demand (BOD) incubator at 37°C under 5% CO2 and 95% humidity. In order to obtain adult worms, female Golden hamsters were injected with 400-500 cercariae. After 45 days, the hamsters were anesthetized with xylazine hydrochloride (10 mg/kg) (Syntec) and ketamine hydrochloride (150 mg/kg) (Syntec) by intramuscular administration. Next, the animals were euthanized with a 2.5% sodium thiopental (150 mg/kg) (Cristália) overdose. For adult worms, perfusion was performed according to Pellegrino and Siqueira (1956). Recovered adult worms were washed four times with Roswell Park Memorial Institute (RPMI) 1640 medium (Gibco, ThermoFisher Scientific) containing 2% penicillin/streptomycin (Gibco, ThermoFisher Scientific). Males and females were separated and incubated for one hour in a BOD incubator at 37°C under 5% CO2 and 95% humidity. After washing, eight males and eight females, separately, were maintained per well in 24-well plates containing 1 mL of RPMI 1640 supplemented with 2% penicillin/streptomycin and 10% inactivated fetal bovine serum. Parasites were incubated for 24 hours in a BOD incubator at 37°C under 5% CO2 and 95% humidity.

### Protein Kinase Structure and *In Silico* Screening

The Protein Homology/analogY Recognition Engine V2.0 (Phyre2) homology modeling platform (Kelley et al., 2015) was used to generate the schistosome kinase protein structures in the absence of resolved crystal structures. The full length predicted amino acid sequences of SmERK1 (Smp_142050), SmERK2 (Smp_047900), SmFES (Smp_332370), SmJNK (Smp_172240), and Smp38 (Smp_133020) without any modification were obtained from *S. mansoni* genome version 7 deposited at the WormBase Parasite database (https://parasite.wormbase.org/Schistosoma_mansoni_prjea36577/Info/Index/) and the human orthologues sequences at the Uniprot database (https://www.uniprot.org). The PDB codes of the human orthologues used for each selected enzyme structure are 1JNK (HsJNK3), 2ZOQ (HsERK1), 5NGU (HsERK2), 3HVC (Hsp38ɑ), and 3BKB (HsFES). Homology models were generated using the default Phyre2 settings in intensive mode. The details for the kinase templates, resolution of the models, and Rwork values are shown in **Supplementary Table S1**. The Ramachandran plot analysis (Ramachandran et al., 1963) was carried out according to Anderson et al. (2005). The resulting homology models and the respective human orthologues were minimized using the Schrödinger Maestro Protein Preparation tool (Schrödinger Release 2021-1: Maestro, Schrödinger, LLC, New York, NY, 2021) on its default settings. The 2D structures of compounds were available as SDF files containing 84666 structures (Library version 8-3-19). These structures were minimized using the Schrödinger Maestro LigPrep tool (Schrödinger Release 2021-1: LigPrep, Schrödinger, LLC, New York, NY, 2021) that also generated protonation states for the pH range 7.0 ± 2.0. The resulting structures were exported as SDF files for use in molecular docking studies. All molecular docking was performed using GOLD V 5.6 Protein-Ligand Docking Software (Jones et al., 1997; Cole et al., 2005) provided by the Cambridge Crystallographic Data Centre (CCDC) on a Linux CentOS workstation. Protein structures were loaded individually into GOLD software and the active sites were defined by the region surrounding the *in situ* ligand. Each ligand was extracted and the docking was carried out using the default parameters of search efficiency and scoring was performed using the PLPscore scoring function, according to Tuccinardi et al. (2010). We limited to create a total of three potential tautomers and ionization states per compound in order to keep the total number of compounds being docked down to a manageable number. The final number of chemical entities docked was roughly around 180,000. Docking was performed for all ten protein structures (five from *S. mansoni* and the five human orthologues). The PDB files for the *S. mansoni* kinase models are available as supplementary files.

### Compounds

The Managed Chemical Compound Collection (MCCC) at the University of Nottingham, United Kingdom, consists of approximately 85,000 unique chemical compounds. The MCCC is a collection of both commercial and in-house research-derived compounds providing a wide range of diverse compounds of variable molecular mass. Most of the compounds in the library were obtained from commercial libraries which were enriched to focus on ‘drug-like’ compounds. The library also includes some compounds synthesized by PhD students and Post Docs at the University. All compounds stored in the library are of >90% chemical purity and most of them obey the Lipinski’s rule of five and have no reactive groups. After the selection of MCCC molecules with predicted affinity for the ATP-binding site of the target kinases, the compounds were ordered and diluted to 5 mM. The dilution was performed using 100% dimethyl sulfoxide (DMSO), ensuring that the final concentration of DMSO to be added to the culture was not higher than 0.4%. The list containing the reference ID and corresponding SMILES of every compound in this study is available in **Supplementary Table S2**.

### *In Vitro* Screening

Compounds were added to schistosomula and adult worm cultures in a final concentration of 20 µM. DMSO 0.4% was used as vehicle control. Two technical replicates were performed for each compound. Compounds that presented significant activity against schistosomula were further evaluated at a final concentration of 10 µM and DMSO 0.2% was used as vehicle control. After 24 and 72 hours of incubation with compounds, schistosomula phenotype was analyzed under an inverted microscope (Axiostar Plus, Zeiss) and the following viability parameters were evaluated: granularity, dark middle region, segmented body, rounded body, and degenerated. A score was given to each well according to the percentage of parasites that exhibited a specific phenotype alteration: 0 when no parasite was affected; 1 when less than 40% of the parasites were affected; 2 when 40-60% of the worms were affected, and 3 when more than 60% of parasites were affected. In parallel, mortality assessment of schistosomula was performed by microscopy by staining with 5 mg/mL of propidium iodide (Merck) and analysis under an Eclipse Ti-E inverted microscope (Nikon) at 10X magnification and 544 nm wavelength. To evaluate the effects of the compounds against the adult stage, worms were incubated with the drugs in the first day and medium was exchanged every second day. The movement of male and female worms was recorded daily for one minute and 30 seconds, for ten days, using the WormAssay software (Marcellino et al., 2012).

### ADMET Predictions

The pharmacokinetic parameters, including the absorption, distribution, metabolism, excretion, and toxicity (ADMET) of compounds that presented antischistosomal activity, were predicted using the pkCSM (http://biosig.unimelb.edu.au/pkcsm/) web server (Pires et al., 2015) and the admetSAR 2.0 (Yang et al., 2019). Then, quantitative data acquired by the pkCSM tool were converted into binary data considering the reference values defined by the program. We also calculated a pkCSM score according to Souza Silva et al. (2021), and an ADMET-score, based on 18 properties predicted by admetSAR 2.0, using a scoring function proposed by Guan et al. (2019). Hierarchical clustering of ADMET binary values was calculated using Euclidean distances using the pheatmap package (v1.0.12) (Kolde, 2015). A comparison between the outcome results of the score analyses was performed and plotted in a Venn diagram.

### Statistical Analysis

Filtering and statistical analysis were performed in R (R Foundation for Statistical Computing, 2018) and GraphPad Prism version 8.0.0 for Windows (GraphPad Software, La Jolla California USA). Mann–Whitney test was used to analyze schistosomula mortality, motility, and surface area. Two-way ANOVA with Dunnett’s multiple comparisons tests was used to analyze adult worms’ motility. Differences were considered significant when p-value < 0.05. Venn diagrams were generated using Venny 2.1 (Oliveros, 2007).

## Results

### Docking-Based *In Silico* Prioritization of Compounds

**Figure 1:**
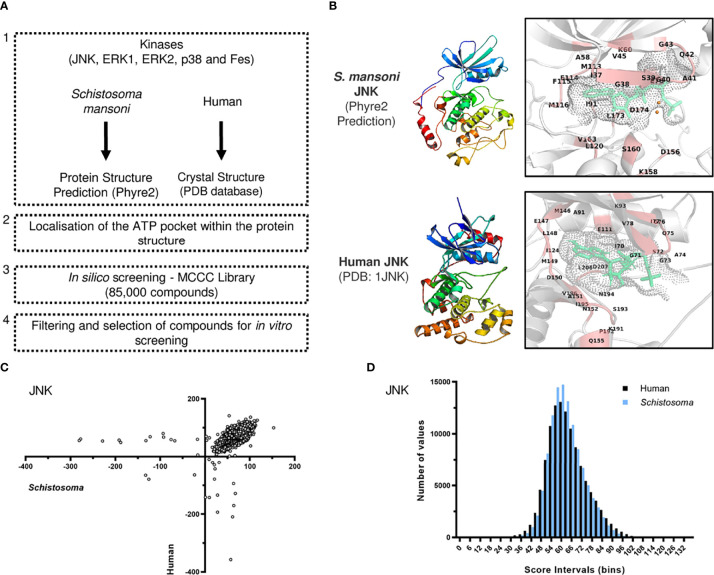
Docking-based *in silico* prioritization of compounds against the ATP pocket of *S. mansoni* protein kinases. **(A)** Workflow for the screening of compounds against the ATP binding pocket of protein kinases from *S. mansoni* and human. **(B)** Ribbon representation of the structure of the homology model (*S. mansoni*) and crystal structure (human, PDB: 1JNK) of JNK protein kinase. The boxes show a close-up view of the ATP pocket with ATP (green) and neighboring residues (red). **(C)** Overall scoring of the docking of the MCCC library of compounds into JNK represented by X-Y plot. Each dot represents one compound and the scores indicate its docking efficiency against the *S. mansoni* JNK (x-axis) and the human JNK (y-axis). **(D)** Histogram representation of the distribution of all scores against *S. mansoni* and human JNK.

Initially, we developed a workflow for selecting compounds based on docking analysis against the ATP-binding pocket of SmJNK, Smp38, SmERK1, SmERK2, and SmFES kinase proteins (**Figure 1A**). We used (1) available crystal structures of the human kinases (PDB database) and the homology models of *S. mansoni* protein kinases in order to (2) localize and (3) screen the ATP binding pocket of the kinases against all compounds from the MCCC library. (4) Then, the best-hit candidates would be selected for further *in vitro* screening (**Figure 1A**). Given the conserved nature of the catalytic domain of the kinase family, we could successfully predict and localize the ATP-binding pocket of the *S. mansoni* kinase protein structures (**Figure 1B** and **Supplementary Figure S1**). Models were generated based on their nearest homologs in the Phyre2 server. Although no specific validation was performed, the confidence scores for the different kinases are reported in **Supplementary Figure S1**, where it can be seen that between 83% and 98% of the residues were modeled with more than 90% confidence (**Supplementary Figure S1**). If we consider only the kinase domain, the confidence was 100% for all five kinases using the corresponding templates covering this domain, according to Phyre2 reports. This is not surprising in view of the high conservation of the overall fold amongst protein kinase domains, and the availability of many crystal structures for their human counterparts in the PDB structural database. This translates also to a high confidence for the modeling of the ATP pocket of each of the kinases, which is known to have a high degree of conservation regarding the characteristics of key amino acids and residues that shape the pocket. The PDB structures of the homology models were subjected to analysis by Ramachandran plots. All of them show a high degree of preferred conformations, similar to the corresponding human orthologs used as templates (**Supplementary Figure S2**). Overall, we screened approximately 85,000 compounds against each pair of the selected kinases (from human and *S. mansoni*): JNK, p38, ERK1, ERK2, and FES. The resulting list with the values for the docking of all compounds was used for ranking and selection of best-hit candidates. The output data from the *in silico* screening generated a large list of scores representing how efficiently a compound docked into the ATP pocket of a given kinase. For a better visualization of the overall profile of the docking for each selected kinase, the scores were plotted to allow a ‘parasite vs human’ comparison for best-hit candidates (**Figure 1C**). In summary, a high score indicates a better docking into the ATP pocket of the kinase, either the human (y-axis) or the parasite one (x-axis) (**Figure 1C**). A histogram was also generated to help visualize the overall distribution of scores among the library of compounds (**Figure 1D**). Apart from ERK2, the overall distribution of the docking scores against the parasite kinases and the corresponding human orthologues were very similar between the selected targets (**Supplementary Figure S3**).

Next, the list of compounds was filtered out in order to narrow down the number of compounds for *in vitro* screening. Our first strategy was to select the highest scored compounds using two approaches. First, we selected the top100-scoring compounds against each of the selected *S. mansoni* kinases regardless of the respective docking values of the same compounds against the human targets (**Supplementary Figure S4A**). We also selected the top100-scoring compounds with preferential binding to the *S. mansoni* kinases when compared to the respective human orthologues (**Supplementary Figure S4A**). In this case, we sought a higher specificity of the compounds towards the parasite kinase. These two approaches provided two distinct lists with a low number of overlapping compounds, apart from FES (**Supplementary Figure S4B**). As second strategy, we prioritized selecting compounds with a more promiscuous activity, i.e., less selectivity at targeting all the selected MAP kinases at the same time, even if this would mean having a lower specificity overall. Therefore, we performed k-means clustering to group compounds with similar score profiles against the MAP kinases (**Supplementary Figures S4C–F**). This resulted in the selection of compounds with a higher score for docking against each of the *S. mansoni* kinases, whilst having a lower score against the corresponding human orthologues. These compounds were termed Pan-kinase inhibitors since we later observed that they also presented a high docking score for the FES kinase. The term Pan-kinase is introduced here to distinguish promiscuous from mono-specific kinase inhibitors. It is not intended to state that these inhibitors would target other, untested protein kinases.

The filtered compounds from the above-mentioned approaches and the promising hits selected for downstream analysis are listed in **Supplementary Table S2**. In total, 169 compounds were prioritized for further *in vitro* screening. From those, 9 compounds specific for SmJNK (SmJNK_1 to SmJNK_9), 15 for SmERK1 (SmERK1_1 to SmERK1_15), 11 for SmERK2 (SmERK2_1 to SmERK2_11), 15 for Smp38 (Smp38_1 to Smp38_15), 28 for SmFES (SmFES_1 to SmFES_28), and 91 predicted to target all kinases (SmPank_1 to SmPank_91).

**Figure 2:**
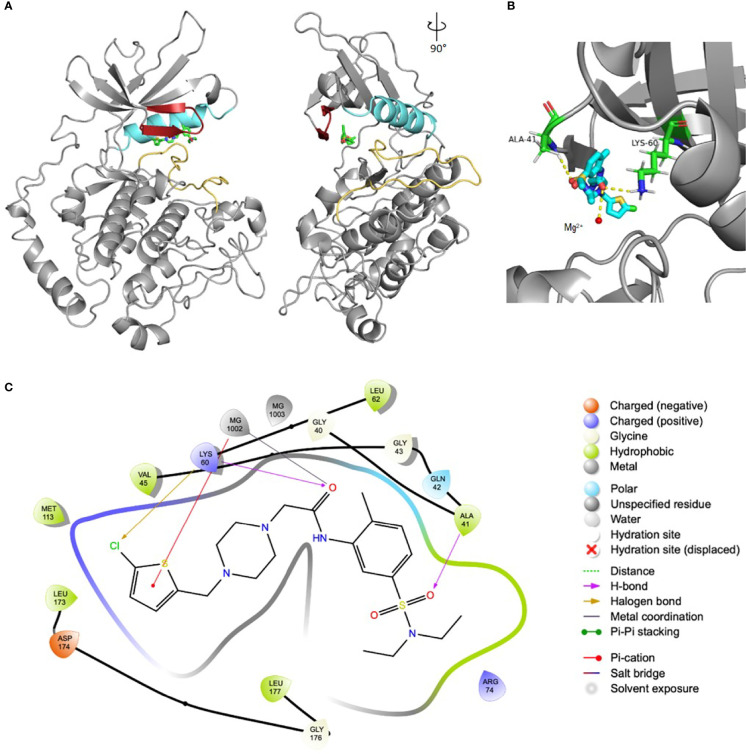
Docking pose of SmPank_12 within the ATP-pocket of SmJNK. **(A)** Ribbon representation of the structure of SmJNK homology model and the ligand SmPank_12. The image was rotated 90° for additional visualization. The main domains that define the ATP-pocket are highlighted in colors: G-loop (red), αC-helix (cyan), and activation loop (yellow). SmPank_12 is represented by sticks. Reference structure: carbon = green; oxygen = red; nitrogen = dark blue; sulfur = yellow; chlorine = dark green. **(B)** Detailed view of the docking site of SmPank_12 as shown in **(A)**. Side chains of Ala41 and Lys60 have polar interactions with SmPank_12 and are represented by sticks. Reference colors of side chains: carbon = cyan; oxygen = red; nitrogen = dark blue. One of the Mg^2+^ is represented by a yellow rounded shape. **(C)** Schematic map represents the detailed interactions of the compounds SmPank_12 within the ATP-binding pocket.

These 169 compounds were predicted to have the highest scores given the corresponding strategies for selection, i.e. displaying the best possible docking pose inside the ATP-pocket of the kinase target. As a means to verify that, we have chosen one of the compounds as an example in order to depict its binding mode and interactions. The protein:ligand interactions between SmJNK and SmPank_12 are shown in **Figure 2**. As expected, SmPank_12 occupies the inner area of the ATP-pocket of SmJNK just between the G-loop domain and αC-helix above the activation loop (**Figure 2A**), similarly to what it is expected to happen when the natural ligand ATP binds to the target kinase. A detailed look of the pocket highlights the interaction of the ligand with one of the Mg^2^+ atoms and the side chains of amino acids Ala41 and Lys60 inside the ATP-pocket (**Figure 2B**). Additionally, a map depicting all main interactions of SmPank_12 within the pocket is shown in **Figure 2C**, where one of the Mg^2+^ ions in the active site is predicted to coordinate the amide carbonyl; this ion also makes an additional π-cation interaction with the thiophene ring. Moreover, the chlorine substituent of the thiophene ring engages with the Lys60 residue through a halogen bonding interaction, and a final hydrogen bond is observed between the sulphonamide and A41 in the protein backbone. Taken together, these observations suggest that the predicted dockings of the selected set of compounds are in line with what is observed for the natural ligand ATP.

### *In Vitro* Screening of Schistosomula

**Figure 3:**
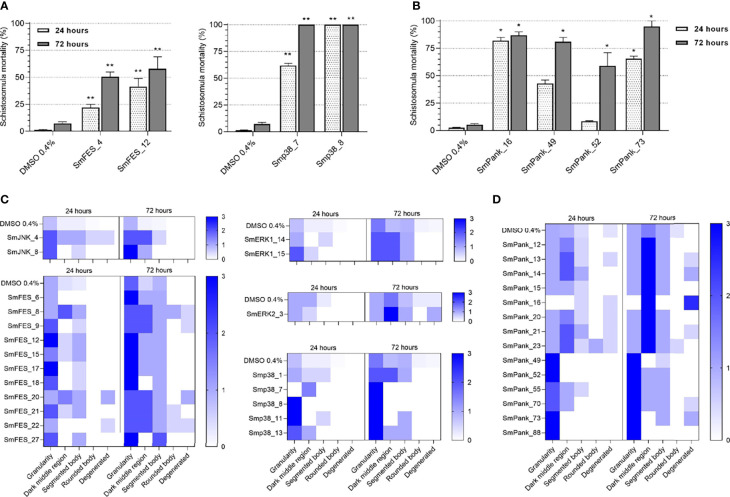
*In vitro* screening of schistosomula identifies 36 active molecules. **(A, B)** Mortality of schistosomula exposed to prioritized MCCC compounds predicted to target single **(A)** or multiple kinase targets **(B)**. Bar graphs representing the mortality mean (%) of schistosomula exposed for 24 hours (white) and 72 hours (grey) to DMSO 0.4% (vehicle control) or to compounds (20 µM). Error bars are represented above the bars. Statistical analyses using Mann–Whitney test are represented with asterisks above the bars (*p < 0.05; **p < 0.01). **(C, D)** Heatmap representation of the mean score given to schistosomula exposed to DMSO 0.4% (vehicle control) or compounds (20 µM) predicted to target single kinases **(C)** or multiple targets **(D)** after 24 and 72 hours of treatment. Schistosomula phenotypes were quantified according to the following parameters: granularity, dark middle region, segmented body, rounded body, and degenerated. The scores ranged from 0 (white) to 3 (dark blue).

Next, we performed *in vitro* screening of all selected compounds against schistosomula. In order to evaluate compound efficacy and assess the parasite viability, we used propidium iodide staining and microscopy analysis. Compounds were considered active when at least 50% in parasite mortality was observed. In total, 8/169 compounds displayed schistosomicidal effects at 20 µM concentration when using this method (**Figure 3A, B**). None of them was predicted to solely target either SmJNK, SmERK1, or SmERK2. Instead, two SmFES-targeting compounds, SmFES_4 and SmFES_12, promoted after 72 hours 50.5% and 58% worm mortality, respectively (**Figure 3A**). Two Smp38-targeting compounds, Smp38_77 and Smp38_8, were highly active as early as 24 hours after exposure causing 62% and 100% mortality, respectively (**Figure 3A**). Moreover, four pan-kinase-targeting compounds were active, promoting a high mortality rate after 72 hours of treatment. The corresponding compounds, SmPank_16, SmPank_49, SmPank_52, and SmPank_73 caused 87%, 81%, 59%, and 95% worm mortality, respectively (**Figure 3B**).

Complementarily, we performed a phenotypic screening of treated schistosomula in order to detect and track morphological alterations of the worm body, which also indicates that a compound can lessen the viability of the parasite. The different phenotypes evaluated are referenced in **Supplementary Figure S5**. By this method, compounds were considered active when the parasites received at least one ‘score 2’ in two or more evaluated parameters or one ‘score 3’ in at least one parameter by visual analysis (see Methods). In total, 35/169 compounds were considered active with 33 of them affecting worm granularity to some extent after 24 hours of treatment (**Figures 3C, D**). This effect was exacerbated during prolonged treatment, i.e., 72 hours of drug exposure, with additional morphological defects becoming more prominent at this later time point, such as the presence of dark-middle region, body segmentation, and body degeneration (**Figures 3C, D**). Amongst the single kinase-targeting compounds, 2/9 were positive for SmJNK, 11/28 for SmFES, 2/15 for SmERK1, 1/11 for SmERK2, and 5/15 for Smp38. The compounds with early strong effects, which are the ones that scored highest for any of the observed parameters at 24 hours, include Smp38_8, Smp38_11, SmFES_12, and SmFES_17 (**Figure 3C**). Regarding the multiple kinase-targeting compounds, a total of 14/91 caused morphological alterations of the worms with four of them causing severe effects 24 hours after treatment, SmPank_49, SmPank_52, SmPank_73, and SmPank_88 **(** **Figure 3D**).

**Figure 4:**
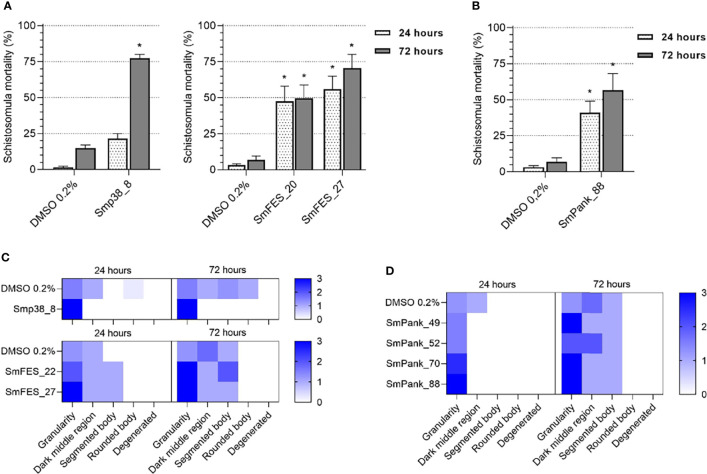
*In vitro* screening of schistosomula identifies eight potent compounds. **(A, B)** Mortality of schistosomula exposed to prioritized MCCC compounds predicted to target single **(A)** or multiple kinase targets **(B)**. Bar graphs representing the mortality mean (%) of schistosomula exposed for 24 hours (white) and 72 hours (grey) to DMSO 0.2% (vehicle control) or to compounds (10 µM). Error bars are represented above the bars. Statistical analyses using Mann–Whitney test are represented with asterisks above the bars (*p < 0.05, , **p < 0.01). **(C, D)** Heatmap representation of the mean score given to schistosomula exposed to DMSO 0.2% (vehicle control) or compounds (10 µM) predicted to target single kinases **(C)** or multiple targets **(D)** after 24 and 72 hours of treatment. Schistosomula phenotypes were quantified according to the following parameters: granularity, dark middle region, segmented body, rounded body, and degenerated. The scores ranged from 0 (white) to 3 (dark blue).

Additionally, in order to identify the most potent compounds, we also screened the previously active compounds at 10 µM. 8/35 compounds showed potent activity at 10 µM (**Figure 4**). Smp38_8 showed significant activity at the 72-hour time point, whereas SmFES_20 and SmFES_27 caused significant schistosomula mortality (around 50%) at 24 hours post-exposure (**Figure 4A**). Although 14/91 Pan kinase-targeting compounds were active at 20 µM, only SmPank_88 caused high schistosomula mortality (56.5%) at 10 µM, after 72 hours of incubation (**Figure 4B** **)**. Additionally, Smp38_8, SmFES_22, SmFES_27, SmPank_49, SmPank_52, SmPank_70, and SmPank_88 caused significant phenotypic alterations of the larvae with predominant “granularity” defects at 10 µM after 72 hours of treatment (**Figures 4C, D**).

### *In Vitro* Screening of Adult Worms

**Figure 5:**
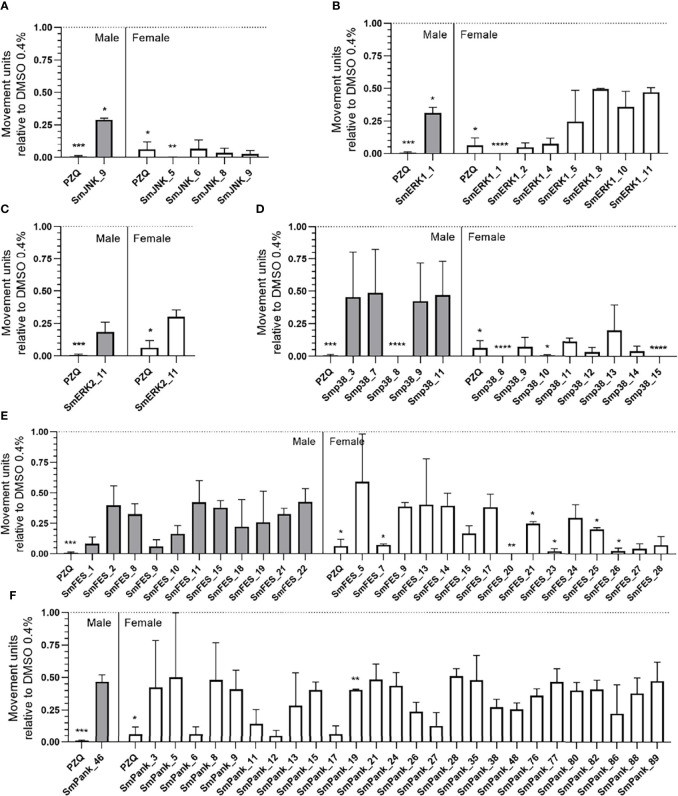
*In vitro* screening of adult worms identifies 73 active molecules. Motility of male (grey bars) and female (white bars) adult worms exposed to prioritized MCCC compounds. Bar graphs represent the mean of the movement units of adult worms exposed to 20 µM of compounds with predicted binding affinity for the ATP-binding site of **(A)** SmJNK, **(B)** SmERK1, **(C)** SmERK2, **(D)** Smp38, **(E)** SmFES, and **(F)** SmPank after ten days of treatment. Praziquantel (PZQ) at 1 µM was used as a positive control. The movement units of worms are plotted relative to the movement units of control adult worms exposed to the vehicle control DMSO 0.4% (dotted line). Error bars are represented above the bars. Statistical analyses using Two-Way ANOVA with Dunnett Multiple comparisons test are represented with asterisks above the bars (*p < 0.05, **p < 0.01, ***p < 0.001 and ****p < 0.0001).

In order to track the effect of compounds in *S. mansoni* adult stage the motility of female and male worms was assessed over ten days. Compounds that provoked at least 50% of motility reduction on the tenth day were considered active. All movement units relative to DMSO 0.4% from adult worms exposed to the compounds are described in **Supplementary Table S3**. For every group of kinase-targeting compounds, female worms were equally or by a superior number of compounds more affected than males (**Figure 5**). 4/9 of the SmJNK-targeting compounds were active, one against both sexes and three against females only. SmJNK_9 exhibited a motility reduction of 71% and 97.4% against male and female worms, respectively. In contrast, SmJNK_5, SmJNK_6, and SmJNK_8 induced a motility decrease of up to 90% in females only (**Figure 5A**). A similar outcome was observed for SmERK1-targeting compounds, where only 1/15, SmERK1_1, was active against both sexes, inducing 100% and 68.9% motility reduction in females and males, respectively. In contrast, 6/15 inhibitors were active only against female adult worms (**Figure 5B**). Moreover, only SmERK2_11 was considered active in its group (1/11), causing reduced motility of female and male worms in 69.8% and 81.7%, respectively (**Figure 5C**). Compounds predicted to bind to Smp38 were also active against adult worms. Smp38_3 and Smp38_7 (2/15) affected exclusively male worms, while 5/15 compounds, Smp38_10, Smp38_12, Smp38_13, Smp38_14, and Smp38_15 affected only females, impairing more than 95% of motility (**Figure 5D**). Furthermore, around 82% (23/28) of the molecules predicted to target SmFES were active against adult worms. SmFES_7, SmFES_20, SmFES_23, and SmFES_26 reduced by more than 90% the motility of female worms after ten days of treatment (**Figure 5E**). Additionally, 3/28 SmFES-predicted inhibitors (SmFES_9, SmFES_15, and SmFES_21) affected both sexes, with SmFES_9 presenting higher activity in male worms (61% motility reduction) after the third day (**Figure 5E**). It is worth mentioning that all female worms exposed to SmFES_20 died on the first day after drug exposure, while no male worms were affected (**Figure 5E**). Regarding the multiple kinase-targeting inhibitors, 27/91 affected the motility of adult worms. Whereas SmPank_46 was active only against males, the remaining 26 were active only against females (**Figure 5F**). Moreover, females exposed to SmPank_6, SmPank_12, and SmPank_17 exhibited more than 93% of motility reduction (**Figure 5F**).

**Figure 6:**
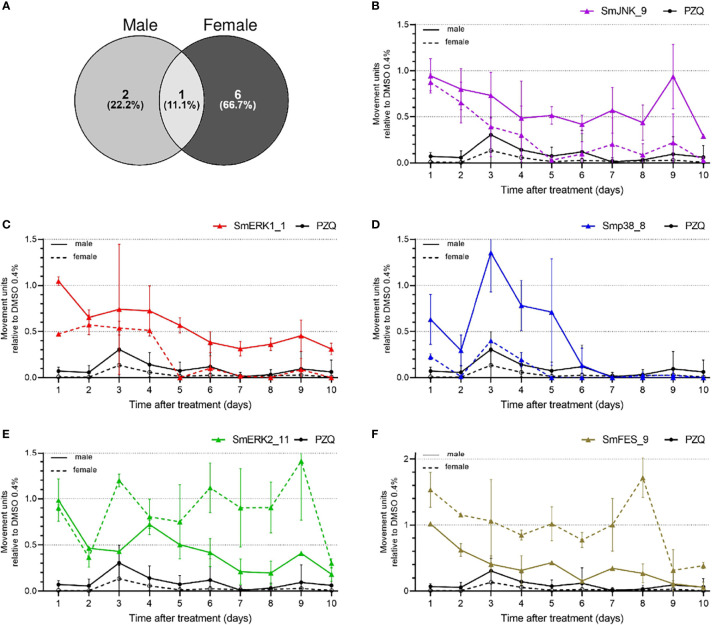
Active compounds affect male and female worms differently. **(A)** Venn diagram representing the number of compounds that presented higher activity when comparing male and female worms. Graphs represent the mean of the movement units of female adult worms exposed to compounds at 20 µM during ten days. Male (solid line) and female (dotted line) adult worms were exposed to **(B)** SmJNK_9, **(C)** SmERK1_1, **(D)** Smp38_8, **(E)** SmERK2_11, or **(F)** SmFES_9 and PZQ at 1 µM was used as reference. Movement units of worms exposed to the compounds are relative to the movement units of worms exposed to the vehicle control DMSO 0.4% (dotted line). Error bars are represented above the bars.

Furthermore, we took a detailed look at the data generated by the WormAssay software, in which we were able to compare, over the course of ten days, how the compounds affected male and female adult worms in comparison to PZQ. Although most of the compounds affected only one of the sexes (**Figure 5**), 9/73 compounds were capable of affecting both male and female worms (**Figure 6**). While two compounds showed a stronger effect against male worms, six presented a stronger activity towards females, and only one equally impaired both sexes motility (**Figure 6A**). Looking closely, SmJNK_9 was able to significantly reduce female movement by the fifth day of treatment, though with a minor effect in males (**Figure 6B**). SmERK1_1 presented a similar effect as observed for SmJNK_9, with females being incapable of moving by the fifth day, unlike males **(** **Figure 6C**). In addition, Smp38_8, which was very strong in killing schistosomula, showed a strong effect both against female and male worms, though in females the stronger effect was observed right from the first day of exposure (**Figure 6D**). In contrast to the previous observations, SmERK2_11 and SmFES_9 reduced rather the male worm movement than the female (**Figures 6E, F**). Other compounds such as Smp38_9, Smp38_11, SmFES_15, and SmFES_21 also affected predominantly female worms by the tenth day, though with similar potency as shown against males in the early time points of the treatment (**Supplementary Figure S6**).

### Active Compounds Against *S. mansoni* and ADMET Predictive Assay

A summary of the number of active compounds for each of the targeted kinases is shown in **Table 1**. Overall, we identified 89 (52.6%) active compounds within our set of 169 prioritized molecules that were selected from the *in silico* analysis. We detected 36/89 active compounds by the schistosomula-screening assay and 73/89 by the WormAssay analysis, whereas 20/89 were active against schistosomula and adult worms (**Table 1** and **Supplementary Table S2**). Moreover, there were more active compounds against female (62/89) worms than against males (20/89) (**Table 1**). Although we had fewer hits (18.2%) among the SmERK2-targeting compounds, the SmFES *in silico* prediction yielded an extremely satisfactory number of active compounds (92.8%). Considering our alternative strategy, where we aimed at selecting compounds that presented good predicted activity against multiple kinase targets, we detected 36/91 (39.5%) hits in at least one of the tests (**Table 1**).

**Table 1:** Summary of the number of compounds selected for *in vitro* analysis for each target kinase.

Target Kinases	Number of prioritized compounds	Number of active compounds	Active compounds per assay			
			Schistosomula	Adult worms		
				Female	Male	Both
SmJNK	9	5	2	3	0	1
SmERK1	15	9	2	6	0	1
SmERK2	11	2	1	0	0	1
Smp38	15	11	5	5	2	3
SmFES	28	26	12	13	8	3
SmPank	91	36	14	26	1	0
**Total**	**169**	**89**	**36**	**53**	**11**	**9**

The number of active compounds is discriminated among assay type and worm sex.

*In silico* predictions including physicochemical, molecular, pharmacokinetic, and toxicity parameters were performed using the computational tool pkCSM (Pires et al., 2015) and admetSAR 2.0 (Yang et al., 2019). ADMET properties and the respective scores for each compound are listed in **Supplementary Table S4**. All the active compounds, except SmFES_6, SmFES_12, Smp38_8, and SmPank _88 obeyed all Lipinski’s rule of five, such as logP < 5, MW < 500 Da, HBA < 10, and HBD < 5 (**Supplementary Table S4**) (Lipinski et al., 1997). The drug-like properties of the compounds were assessed by calculating a score based on the total number of positive features evaluated by pkCSM (**Supplementary Figure S7** and **Supplementary Table S4**), as described in the Methods section. Compounds Smp38_3 and SmFES_6 scored the highest, 17, regarding drug-like properties (varying from 1 to 17 for the active compounds). In total, 29/89 compounds scored equally or higher than the known schistosomicidal drug PZQ, which scored 11 (**Supplementary Table S4**). In addition, the ADMET predictions obtained using admetSAR 2.0 were used to calculate an ADMET-score (**Supplementary Figure S8** and **Supplementary Table S4** **)**. This analysis revealed 40/89 compounds with scores higher than PZQ (0.693). Compound SmPank_9 scored the highest (0.920) considering the 18 drug-like properties (varying from 0.920 to 0.487 for the active compounds). The comparison between the two approaches revealed 17/89 compounds presented good scores in both approaches (**Supplementary Figure S9**). Although this indicates that many of the selected hits have desirable drug-like properties, most of them should be further optimized before using in subsequent *in vivo* assays in order to avoid undesired toxicity and side effects, since a few (7 considering the pkCSM prediction and 11 in admetSAR 2.0 prediction) were not considered hepatotoxic (**Supplementary Table S4**).

## Discussion

Traditional High-Throughput Screening (HTS) is considered a standard method for discovering multiple hit compounds. However, the hit rate is very low and dependent on the screening of large compound libraries (Sundberg, 2000). To counteract that, Computer-Aided Drug Design (CADD) has emerged as an approach to significantly boost the drug discovery process. One of the main purposes of using CADD is to filter large compound libraries and decrease the number of molecules necessary to be experimentally tested, while still being able to identify hits with the same efficiency (Sliwoski et al., 2014). In structure-based CADD, target structure and ligand docking methods can substantially reduce the cost and workload of HTS, which makes the use of both strategies in parallel a valuable tool. Here, we have employed CADD to identify active lead compounds against five *S. mansoni* protein kinases. The strategy used in this study allowed the prioritization of 169 compounds from the MCCC library, of which 89 (52.6%) were considered active in *in vitro* screening assays by causing either reduced viability and/or morphological defects in larval stages, or reduced motility in adult worms. The number of identified active compounds using the aforementioned strategy is considered high when compared to similar studies. A seminal study assessed traditional HTS and CADD prioritization, which were employed concurrently against two large compound libraries when searching for inhibitors against the protein tyrosine phosphatase-1B (PTP1B) (Doman et al., 2002). Of approximately 400,000 molecules screened by HTS assay, 85 (0.021%) showed inhibitory activity. In contrast, by using molecular docking against the same enzyme structure, the researchers selected 365 molecules out of a 235,000-compound library and a striking number of 127 (34.8%) were considered active in the same assay (Doman et al., 2002). The abovementioned observations highlight the importance of complementary use of strategies in the process for identification of active molecules against known targets, making the search for new drugs more direct and cost-effective.

Our goal was to identify compounds that have high a predicted binding affinity for the ATP-pocket of kinase targets that have been shown to play very important roles in *S. mansoni* development and infection establishment (Andrade et al., 2014; Avelar et al., 2019; Tavares et al., 2020). Hence, we used a rational approach focusing on the ATP-binding site of SmJNK, SmERK1, SmERK2, Smp38, and SmFES to perform an *in silico* screening and select inhibitors with the highest score for docking into the ATP-pocket. In general, the kinase protein family shares a great degree of sequence conservation, and this translates into a highly similar kinase domain, both in structural pocket formation and in common substrate recognition motifs (Manning et al., 2002). Considering the similarity between the ATP-binding sites among different kinases, the challenge lies in whether an identified hit compound can be developed and optimized for a desirable degree of selectivity. Yet, most of the small-molecule kinase inhibitors are classified as type I, i.e., targeting the ATP-binding pocket of the kinase domain (Liu and Gray, 2006). Therewithal, we selected compounds that presented a higher docking specificity towards the *S. mansoni* kinases rather than the corresponding human orthologues. Thus, we expected to favor compounds that would preferentially target the parasite kinases with less off-target effects in future lead development. Because no crystal structure is yet available for the parasite’s kinase orthologues, we relied on comparative homology models of the target proteins; an approach that is fairly common in CADD studies (Becker et al., 2006; Warner et al., 2006; Gaba et al., 2014; Nalini et al., 2018). Since the *S. mansoni* kinases selected for this study are well conserved, the predicted homology models for the parasite’s kinases presented a high degree of confidence, allowing a reliable *in silico* screening using the ATP-binding site.

The initial strategy in drug discovery history was focused on finding potent compounds against a single target protein of interest. Higher specificity was desired in order to avoid off-target effects of candidate drugs (Chartier et al., 2017). However, this goal has proved many times complex and hard to achieve especially due to complex therapeutic effects. Since then, the recent consensus has leaned from desiring a highly selective inhibitor of a single target towards favoring a multi-target selectivity (Hopkins, 2008; Nobeli et al., 2009; Jalencas and Mestres, 2013). Nevertheless, some careful steps should be taken when tackling selectivity vs specificity in drug screenings. One study explored the concept of polypharmacology by investigating the ability of finding compounds with joint activity from a large library of compounds (Weiss et al., 2018). The authors reported that although they could yield a high number of on-target hit rates with good selectivity, these compounds were just as good against the antitargets, even with molecules being selected for their predicted lack of binding to off-targets. Based on the idea that a good selectivity is necessary to balance efficacy and toxicity we employed an alternative strategy for the *in silico* screening. We hypothesized that a drug able to target multiple kinases, especially those that participate in overlapping signaling cascades (e.g., MAP kinases), would be beneficial in terms of inhibition of a redundant signaling pathway. Indeed, RNAi knockdown of several MAP kinase proteins has shown to consistently affect *S. mansoni* development and/or reproduction (Andrade et al., 2014; Avelar et al., 2019; Tavares et al., 2020) and also regulate many genes in common (Gava et al., 2019). Here, we reported 91 compounds predicted to target the five analyzed target kinases, from which 14 and 27 presented activity against schistosomula and adult worms, respectively. Multi-target drugs have better effects when used to treat complex diseases that involve multiple genes and factors. For instance, imatinib is a tyrosine kinase inhibitor known to be active against multiple targets, such as the Abl, Arg, c-KIT, PDGF-R, and the oncogenic form BCR-ABL (Manley et al., 2002; Waller, 2018). The same can be said for later developed 2^nd^ and 3^rd^ generation of tyrosine kinase inhibitors such as dasatinib, nilotinib, bosutinib, and ponatinib (Waller, 2018). Hence, our data and these observations reinforce the importance of also focusing on the concept of polypharmacology, aiming for one drug – multiple targets.

Additionally, visual analysis by microscopy remains the gold standard for *Schistosoma* sp. drug screening (Abdulla et al., 2009; Lombardo et al., 2019). However, some studies have suggested the use of other quantitative methods in parallel to strengthen the observations (Manneck et al., 2011; Aguiar et al., 2017; Maccesi et al., 2019). In order to be stricter, we applied three methodologies in our *in vitro* screening assays: propidium iodide for checking schistosomula viability, and visual analysis and worm assay to assess the motility of adult worms. Accordingly, our data support the need of using at least two screening strategies to gather the most information on possible active molecules. This strategy lowers the likelihood of selecting false negatives, since the compound activity could be stage- or sex-specific and reinforces the idea that compounds active in more than one assay are more likely to be good lead compounds.

Our data revealed that 36 (40.4%) active compounds presented activity against the larval stage of the parasite. Finding alternative therapeutic drugs that tackle the earliest stages of the parasite’s life cycle in the definitive host is necessary. PZQ is the most successful drug used for schistosomiasis treatment, meaning a major advance in the treatment of this disease (Cioli et al., 1995; da Silva et al., 2017; World Health Organization, 2019). Schistosomiasis treatment relies mainly on PZQ, which despite having high potency against all species of *Schistosoma* spp., can present very low efficacy against some life stages of the worm such as 3 to 4-week old larva (Doenhoff et al., 2008), slowing down the eradication of schistosomiasis in endemic areas requiring repeated mass treatments in the same population (Fenwick and Webster, 2006; Melman et al., 2009; Crellen et al., 2016; da Silva et al., 2017). In the context of *in vitro* drug screening, a more recent publication showed that juvenile stages developed in vitro and in vivo have similar sensitivity to PZQ when treated *in vitro* (Buchter et al., 2021), which gives support to the methodology used here. Based on that, candidate lead compounds that show efficacy against schistosomula and/or at least one adult worm sex can be explored to support schistosomiasis treatment, while those that target only the larval stages can be developed for use in combination with PZQ. Likewise, it will be interesting to assess the efficiency of active compounds identified in this study against other species of schistosomes.

*S. mansoni* male and female adult worms exhibit differentiated development and metabolism, and consequently, the susceptibility of each sex to a particular drug is distinct. In fact, males are relatively more susceptible to PZQ than females, although other factors such as worm stage and pairing status do also play a role (Shaw and Erasmus, 1983; Pica-Mattoccia and Cioli, 2004). However, when it comes to our prioritized kinase inhibitors, most of the compounds reported to be active against the adult stage affected female worms preferentially. It is reported that differences in protein content in the tegument, age of the worm, pairing status, and strains can substantially alter the absorption and effect of compounds for each sex in different ways (Shaw and Erasmus, 1983; Shaw, 1990; Pica-Mattoccia and Cioli, 2004; Da’dara et al., 2012; Pinto-Almeida et al., 2016). We hypothesize that the smaller size and thinner tegumental and subtegumental layers of female worms could facilitate permeation and/or the effect of drugs. Although we have not followed in detail the morphological alterations of treated female worms, our data suggest that females are more susceptible than males for this group of prioritized inhibitors. Indeed, the importance of protein MAP kinases in the reproductive biology of *S. mansoni*, especially female worms, has been reported (Andrade et al., 2014; Avelar et al., 2019). These observations are reinforced by studies in which other kinase inhibitors also cause severe effects in female worms. For instance, the *Schistosoma* ABL inhibitor imatinib causes considerable phenotypic changes in the ovary, vitellarium, and gastrodermis of female worms from *S. mansoni* (Beckmann and Grevelding, 2010) and S. *japonicum* (Li et al., 2019). This could shed light in a new direction in the discovery of new drugs capable of impair the reproduction of *Schistosoma* sp. and/or lessen the disease pathology by targeting female worms and their reproductive roles such as in egg production. Nevertheless, two out of the nine compounds that affected both sexes in the present study had a stronger effect on males on the tenth day of exposure to the drugs, which highlights that discovering potent drugs against both sexes is also a viable alternative.

Pharmacokinetic and toxicity properties determine whether it is possible to advance and achieve therapeutic success with lead compounds in the drug development process (Pires et al., 2015). In this context, the ADMET analysis was carried out in our study with all active molecules evaluated by the *in vitro* screenings. This strategy helps to determine which molecules have the best prerequisites to proceed to *in vivo* tests, reducing not only the number of animals used in the experiments, but also time, labor, and costs. Hence, *in vivo* analysis for some active compounds must be postponed or not carried out if, for example, any molecules are predicted to be carcinogenic. Regarding toxicity, most active compounds (92%) were predicted to be hepatotoxic. Indeed, kinase inhibitors, especially tyrosine kinase inhibitors, are known to promote hepatotoxicity during their clinical use (Shah et al., 2013). Some TKI-mediated hepatotoxicity mechanisms include the production of reactive metabolites through the metabolism and bioactivation by cytochrome P450 enzymes. Among the active compounds, 86.3% were predicted as a substrate for CYP3A4, one of the enzymes related to the metabolism of several TKIs, like erlotinib, dasatinib, and lapatinib (Li et al., 2009; Li et al., 2010; Teng et al., 2010). Moreover, 21 (23.8%) active compounds presented MRTD (Maximum Recommended Tolerated Dose) values above 0.477, indicating that those are not likely to cause toxicity in humans. Some properties (e.g., inhibitory concentrations, toxicity in mammalian cells, selectivity index) should be checked *in vitro* before assessing the schistosomicidal effect of compounds *in vivo*. Together, our results show that at least 17 compounds out of the 89 active compounds presented good pharmacokinetic properties (pkCSM score ≥ 11 and ADMET-score ≥ 0.693) and should be prioritized for future *in vivo* tests.

## Conclusion

In summary, this study provides proof of concept that SmJNK, Smp38, SmERK1, SmERK2, and SmFES are promising drug targets for *S. mansoni* treatment and shows the applicability of combining *in silico* screening along with HTS methodologies for the identification of new active molecules targeting *S. mansoni* proteins. The strategy applied here proved to be efficient and considerably increases the likelihood of finding new candidate lead compounds. When it comes to schistosomiasis, methodologies that reinforce the search for drugs that tackle schistosomula or, even better, both stages of the parasite that occur in the human host, can be quite beneficial for reaching the goal of a new and more efficient therapeutic for the disease.

## Data Availability Statement

The raw data supporting the conclusions of this article will be made available by the authors, without undue reservation. Information regarding the Managed Chemical Compound Collection (MCCC) can be obtained by contacting the MCCC Manager, School of Pharmacy, University of Nottingham.

## Ethics Statement

Animals’ procedures were approved by the Ethics Commission on Animal Use (CEUA) of the Oswaldo Cruz Foundation under the number LW-12/16. Experiments were performed under Brazil national guidelines following Law 11794/08.

## Author Contributions

FF and MM designed the study and contributed with reagents, materials and analysis tools; BM and TA performed the computational analysis and the *in silico* screening; IB, NT, SG, GT, and MM performed the *in vitro* experiments; SG performed the ADMET analysis; FF, MM, BM, IB, NT, and SG wrote the manuscript. All authors contributed to manuscript revision, read and approved the submitted version.

## Funding

This work was funded by the LOEWE Centre DRUID within the Hessian Excellence Initiative to FF; Conselho Nacional de Desenvolvimento Científico e Tecnológico (CNPq) (Fellowship Grant number 302518/2018-5 and 317389/2021–1) and FAPEMIG (CBB-APQ-0520-13) to MM; and by the Coordenação de Aperfeiçoamento de Pessoal de Nível Superior–Brasil (CAPES)–Finance Code 001, and CAPES Programme on Drug Discovery - CAPES/Nottingham University (003/2014).

## Conflict of Interest

The authors declare that the research was conducted in the absence of any commercial or financial relationships that could be construed as a potential conflict of interest.

## Publisher’s Note

All claims expressed in this article are solely those of the authors and do not necessarily represent those of their affiliated organizations, or those of the publisher, the editors and the reviewers. Any product that may be evaluated in this article, or claim that may be made by its manufacturer, is not guaranteed or endorsed by the publisher.
