# Supplementary material for: Docking-Based Virtual Screening Enables Prioritizing Protein Kinase Inhibitors With In Vitro Phenotypic Activity Against Schistosoma mansoni
Source: Front Cell Infect Microbiol. 2022 Jul 5;12:913301. doi: 10.3389/fcimb.2022.913301 (PMC9294739; doi:10.3389/fcimb.2022.913301)
Supplement: Supplementary file 1 [file DataSheet_1.docx]

**Supplementary Data**

**Supplementary Table S1: Template information for the selected kinase proteins.** List of PDB templates, with corresponding ligands, and resolution and Rwork values for each of the selected kinases.

| **Protein** | **Template PDB** | **Ligand** | **Resolution / Å** | **Rwork** |
| --- | --- | --- | --- | --- |
| ERK1 | 2ZOQ | 5-fluorotubercidin | 2.39 | 0.233 |
| ERK2 | 5NGU | Small molecule inhibitor | 2.74 | 0.267 |
| P38 | 3HVC | Small molecule inhibitor | 2.10 | 0.226 |
| JNK | 1JNK | ANP – ATP analogue with the beta-gamma phosphate oxygen bridge replaced with a nitrogen | 2.30 | 0.222 |
| FES | 3BKB | Staurosporine | 1.78 | 0.148 |

**Supplementary Table S2: Predicted docking scores for the top100-scoring compounds and the prioritized compounds targeting the selected *S. mansoni* and human protein kinases.** The table is separated into six sheets with the first two showing the top100-scoring compounds selected from strategy 1 depicted in **Supplementary** **Figure 4A-B**, and the third sheet showing the selected compounds from strategy 2 depicted in **Supplementary** **Figure 4C-F**. The fourth sheet lists the set of prioritized compounds for *in vitro* screening and further analysis. The fifth sheet lists the assays and adult worm sex for each of the **89** active compounds. The sixth sheet contains the compound ID and respective SMILES strings for every compound referenced in this study.

**Supplementary Table S3:** **Movement units of adult worms tracked by WormAssay software.** The table depicts the mean of the movement units of female and male adult worms exposed to compounds at 20 µM concentration on the tenth day. Movement units are represented relative to the movement units of female adult worms exposed to the vehicle control DMSO 0.4%.

**Supplementary Table S4: Predicted ADMET properties and druggability score of the active compounds.** The table is separated into four sheets with the first containing the ADMET (Absorption, Distribution, Metabolism, Excretion, and Toxicity) properties predicted by the pkCSM computational tool. The second sheet lists scores of the interpretation of the data on the first sheet based on the pkCSM score proposed by Silva et al. (2021). The score is the total sum of positive features. P, lipophilicity; Papp, apparent permeability coefficient; Kp, skin permeability constant; VDss, volume of distribution; Fu, fraction unbound; BBB, blood-brain barrier; BB, blood-brain; CNS, central nervous system; PS, blood-brain permeability-surface area product; AMES, method to assess a compound mutagenic potential using bacteria; LD, lethal dosage; LOAEL, lowest-observed-adverse-effect level; *T. pyriformis*, *Tetrahymena pyriformis*. The third sheet containing the ADMET properties predicted by the admetSAR 2.0. The fourth sheet lists ADMET-scores based on 18 properties predicted by admetSAR 2.0, using a scoring function proposed by Guan et al. (2019).


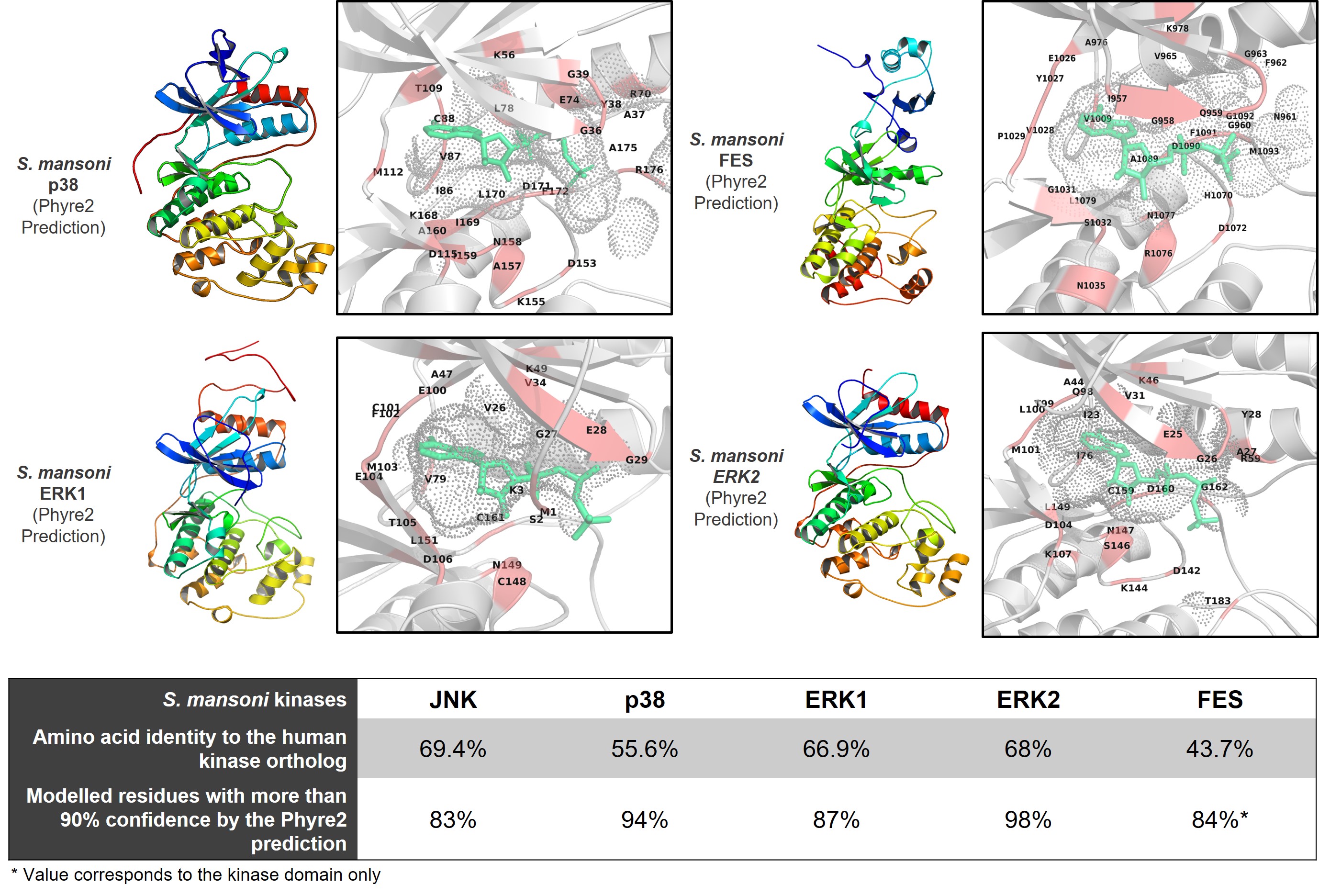


**Supplementary Figure S1: Homology model of *S. mansoni* protein kinases.** Ribbon representation of the 3D-structures of *Schistosoma* p38, ERK1, ERK2, and FES protein kinases predicted by Phyre2 using intensive modeling mode. The boxes depict a close-up view of the ATP pocket with ATP (green) and neighboring residues (red). The table at the bottom shows the identity of the kinases to the human orthologues and the prediction confidence for 90% of the modeled residues. The PDB files corresponding to each selected kinase are available for download separately.


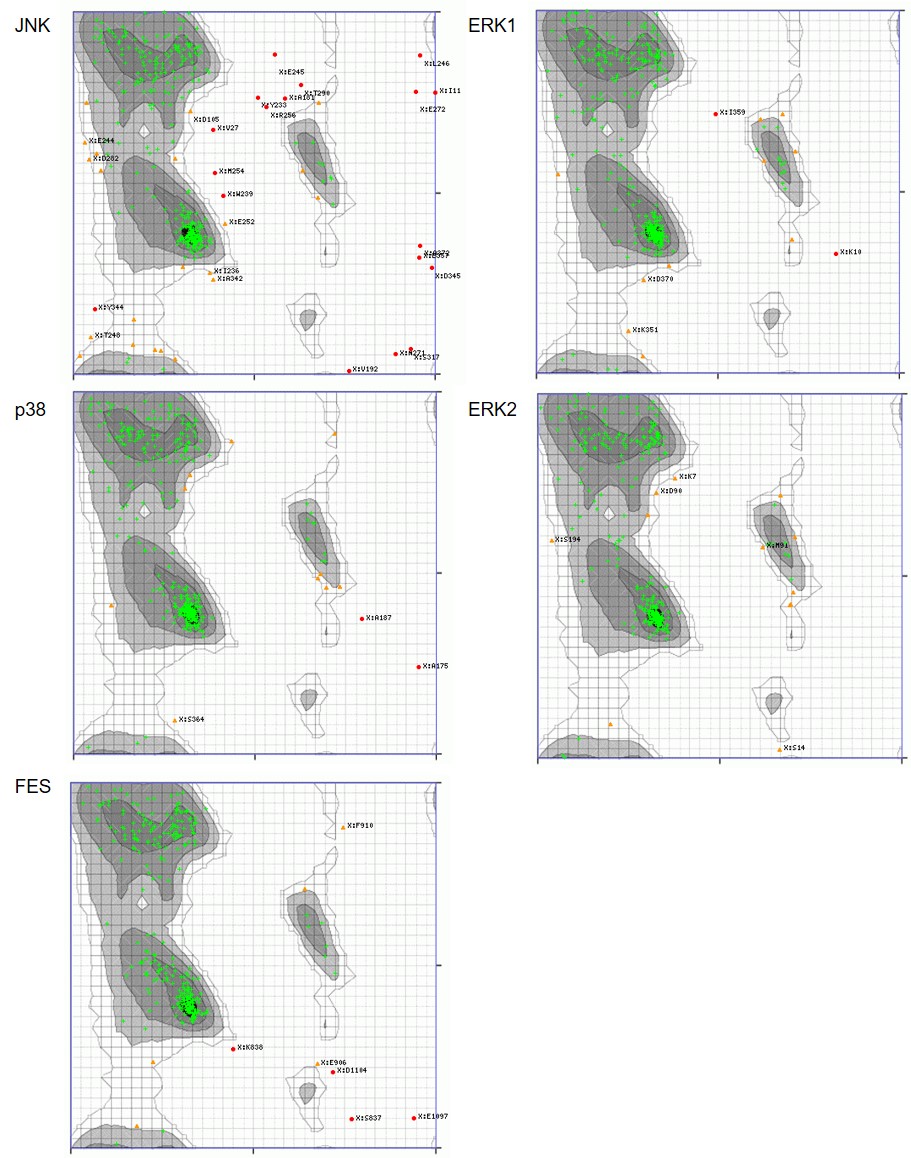


**Supplementary Figure S2:** **Ramachandran plots of the five *S. mansoni* kinases used in this study.** The generated PDB models used for the docking were subject to Ramachandran plot analysis according to Anderson et al. (2005). The chart is color-coded: Black, dark grey, grey, and light grey areas represent highly preferred conformations. Green crosses represent highly preferred observations, orange triangles represent preferred observations, and red circles represent questionable observations from generated models.


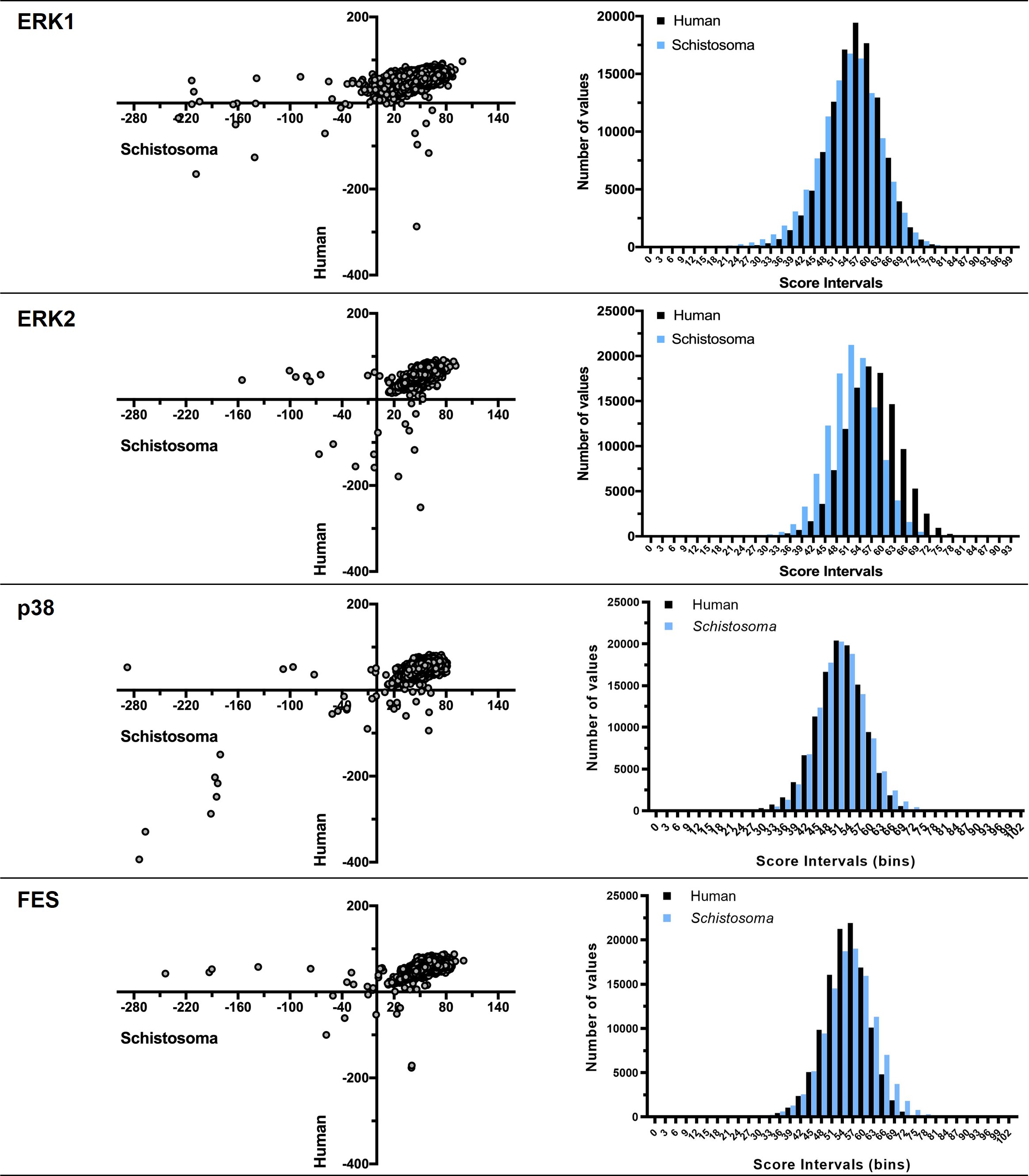


**Supplementary Figure S3:** **Overall scoring profile of the MCCC library of compounds against ERK1, ERK2, p38, and FES protein kinases.** For each pair of kinase targets, the left X-Y plot represents all compounds and their corresponding docking scores against the *S. mansoni* (x-axis) and the human (y-axis) target. On the right, the histogram depicts the distribution of all compound scores for each pair of targets.


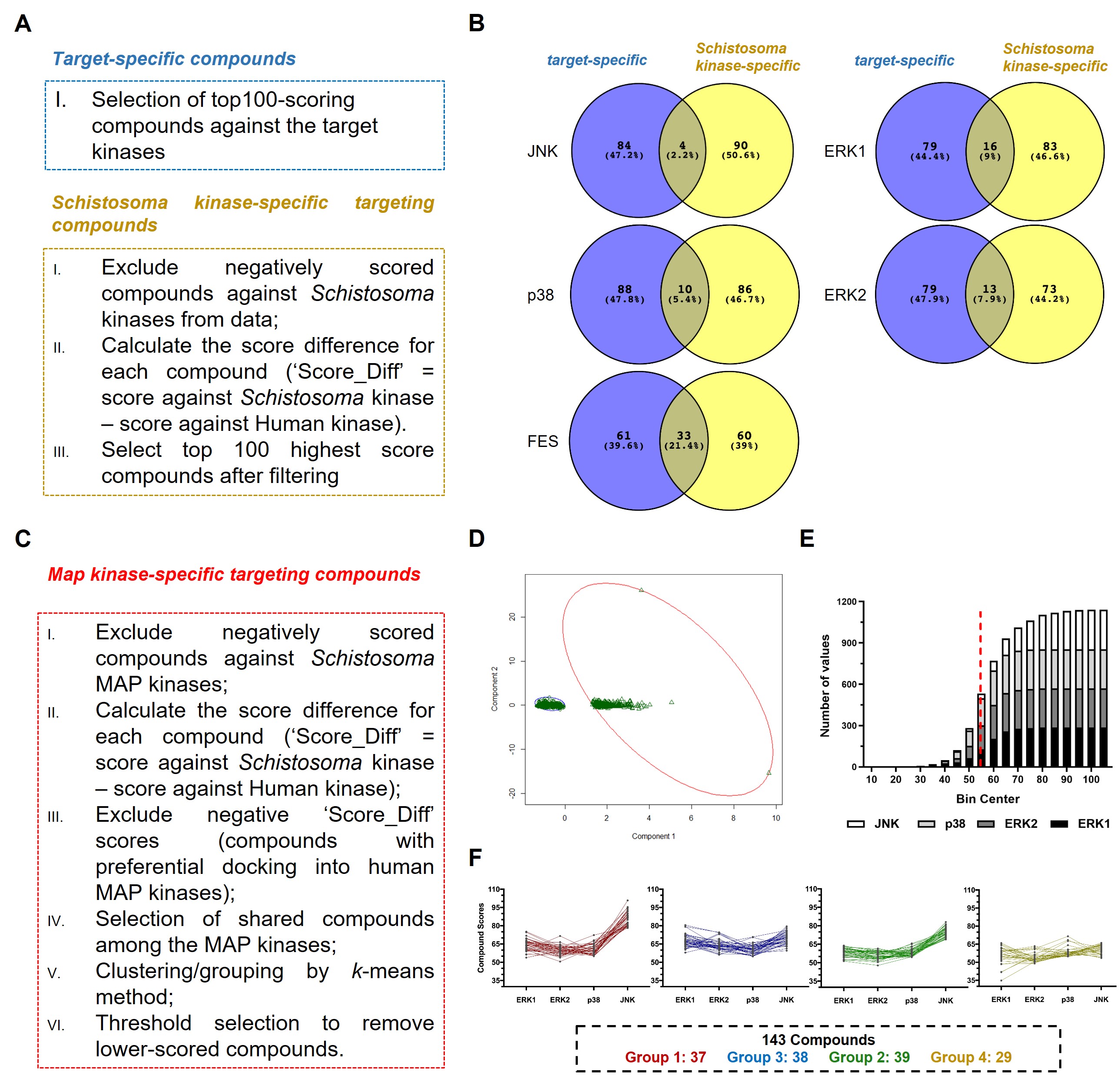


**Supplementary Figure S4:** **Strategies for compound selection against JNK, p38, ERK1, ERK2, and FES kinases.** (A) Compounds were selected either by their target-specific scoring values or by their higher specificity of binding to *S. mansoni* kinases in comparison to the human ortholog. (B) Venn diagrams comparing the list of compounds originated from (A). (C) Workflow of the alternative strategy for the selection of compounds with promiscuous selectivity against *S. mansoni* MAP kinases. (D) Compounds were grouped by k-means clustering and compounds with optimal efficiency score (red circle) were used for further analysis. (E) Frequency distribution was applied to remove compounds with a lower absolute score for targeting MAP kinases. (F) Scoring profile of the remaining 143 compounds against the MAP kinases after the threshold application.


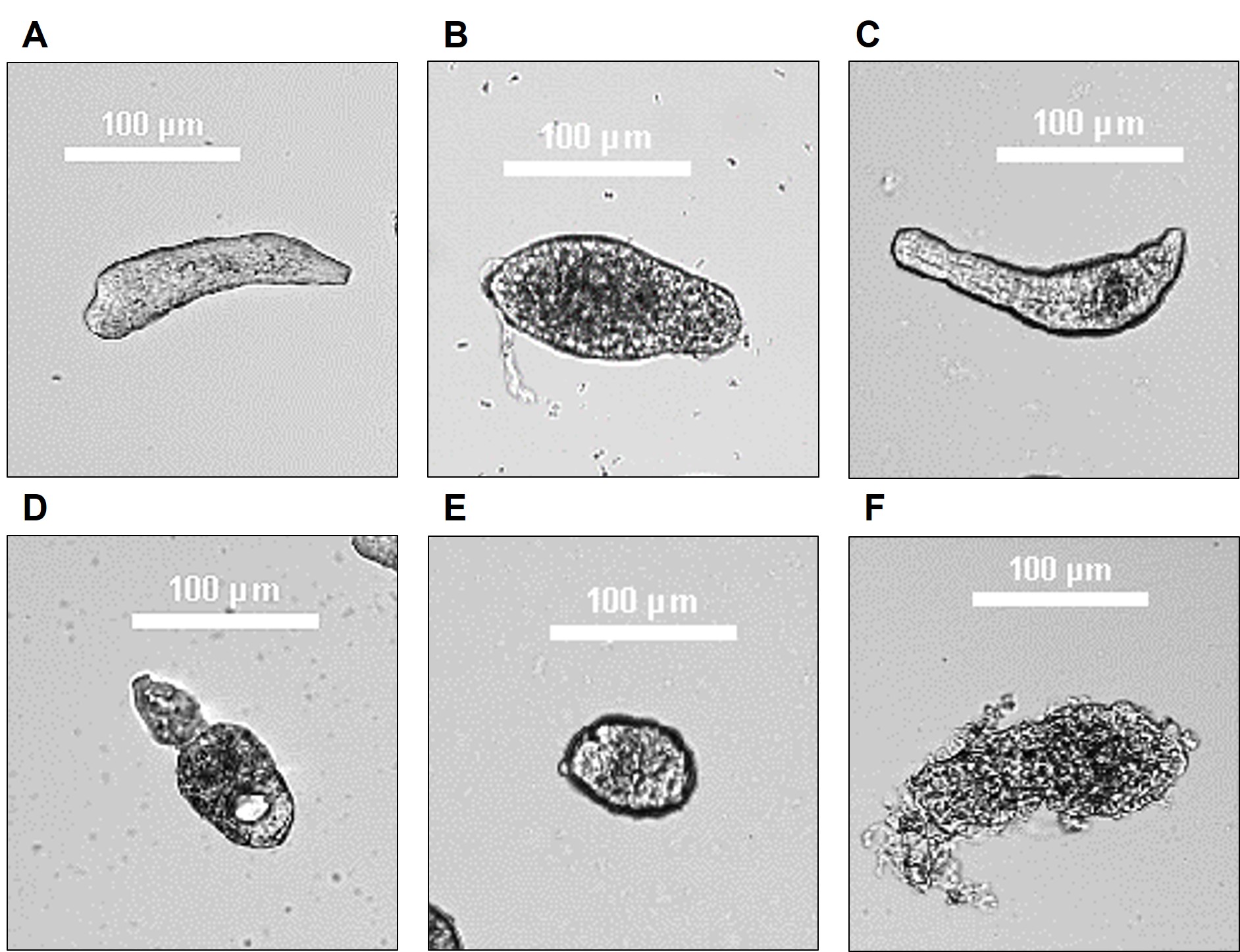


**Supplementary Figure S5: Reference phenotypes for evaluation of schistosomula**. Schistosomula were categorized into six different phenotypes: (A) viable; (B) granularity; (C) dark middle-region; (D) segmented body; (E) rounded body; (F) body degeneration. Compounds were added to a final concentration of 20 µM in duplicate. Parasites were evaluated after 24 and 72 hours of treatment. White bars indicate 100 μm.


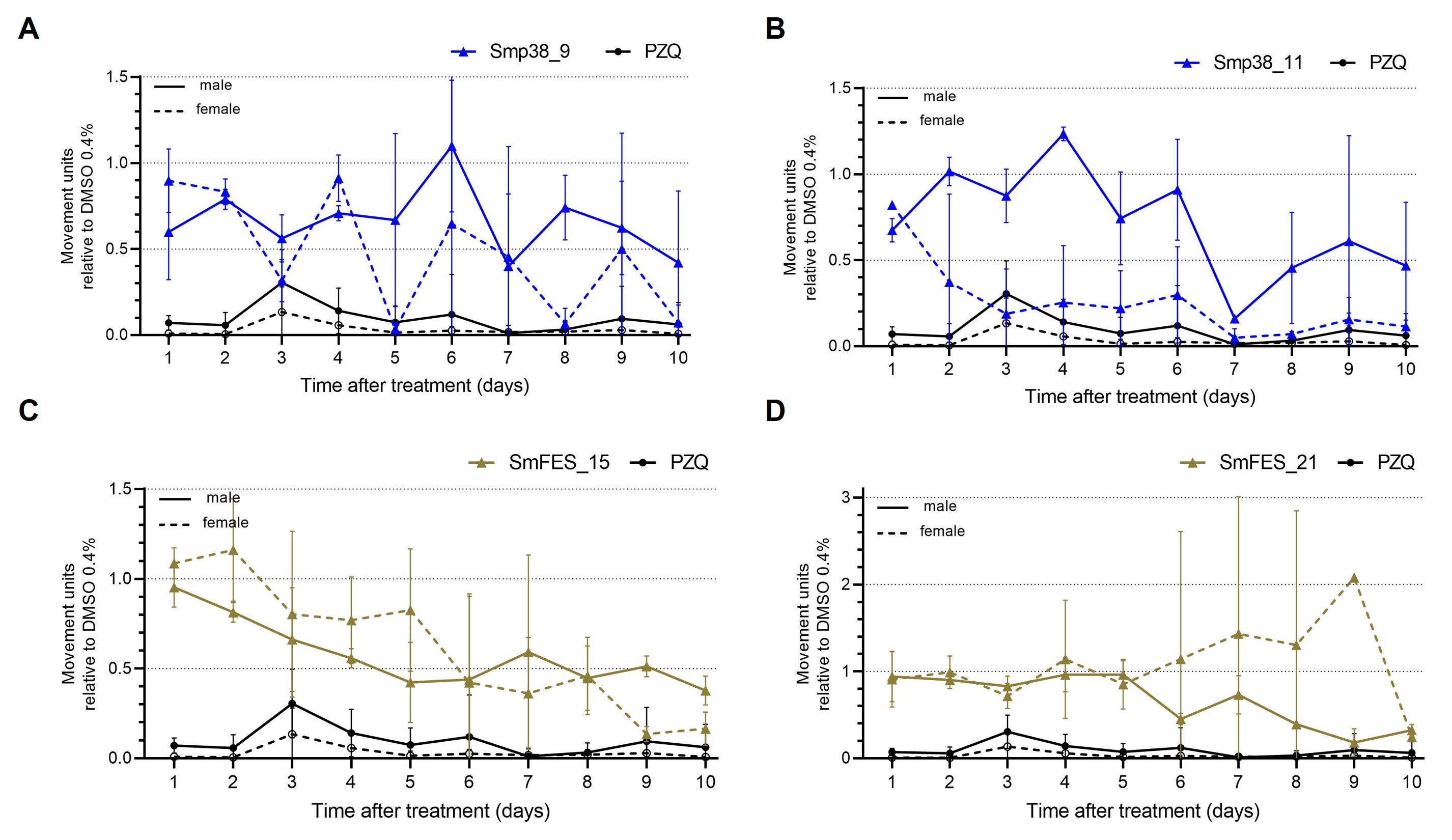


**Supplementary Figure S6: Active compounds affect male and female worms differently.** Graphs represent the mean of the movement units of female adult worms exposed to compounds at 20 µM over the course of ten days. Male (solid line) and female (dotted line) adult worms were exposed to (A) Smp38_9, (B) Smp38_11, (C) SmFES_15, or (D) SmFES_21 or PZQ at 1 µM as reference. Movement units of worms exposed to the compounds are relative to the movement units of worms exposed to the vehicle control DMSO 0.4% (dotted line). Error bars are represented above the bars.


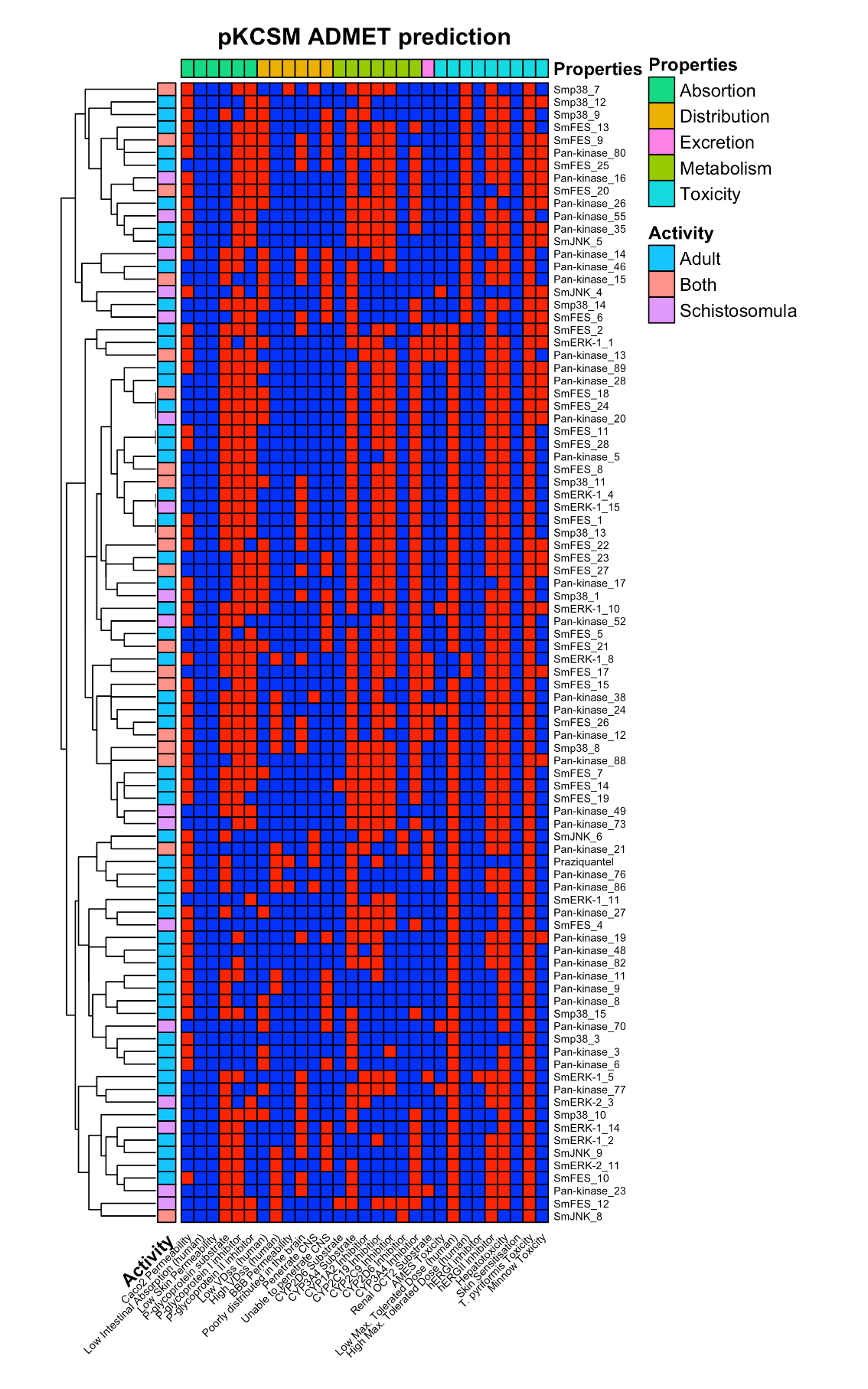


**Supplementary Figure S7: ADMET analysis using the pkCSM prediction algorithm.** Hierarchical clustering of ADMET properties predicted using pkCSM web server. The presence of features desired for good druggability is indicated by red squares while the lack of the feature is represented by blue squares. Column sidebar colors indicate the following ADMET properties: Absorption (green), Distribution (yellow), Metabolism (pink), Excretion (light green), and Toxicity (light blue). The row sidebar colors indicate if the compounds presented activity against adult worms (blue), schistosomula (pink), or both (orange).


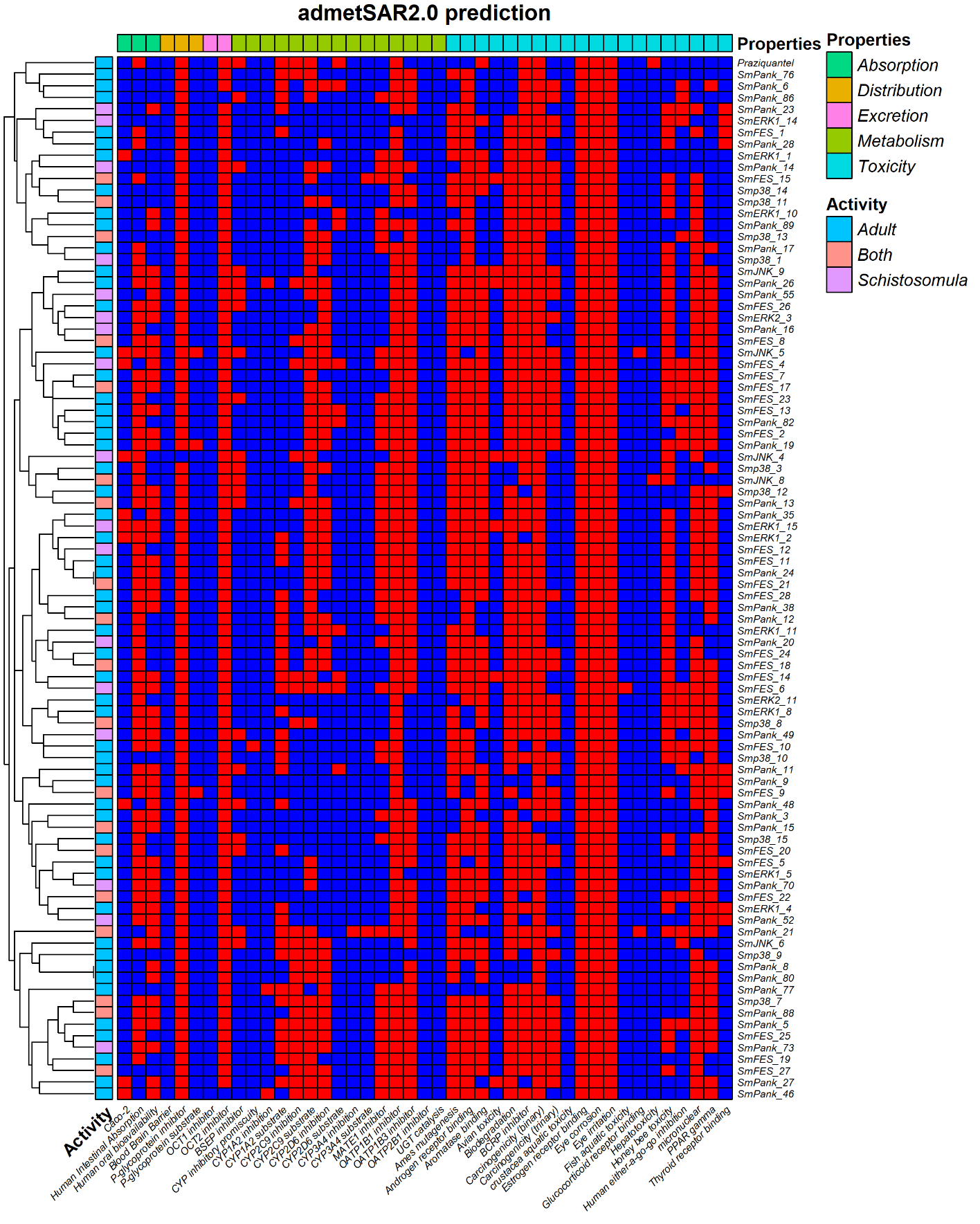


**Supplementary Figure S8: ADMET analysis using the admetSAR 2.0 tool.** Hierarchical clustering of ADMET properties predicted using admetSAR 2.0 web server. The presence of features desired for good druggability is indicated by red squares while the lack of the feature is represented by blue squares. Column sidebar colors indicate the following ADMET properties: Absorption (green), Distribution (yellow), Metabolism (pink), Excretion (light green), and Toxicity (light blue). The row sidebar colors indicate if the compounds presented activity against adult worms (blue), schistosomula (pink), or both (orange).


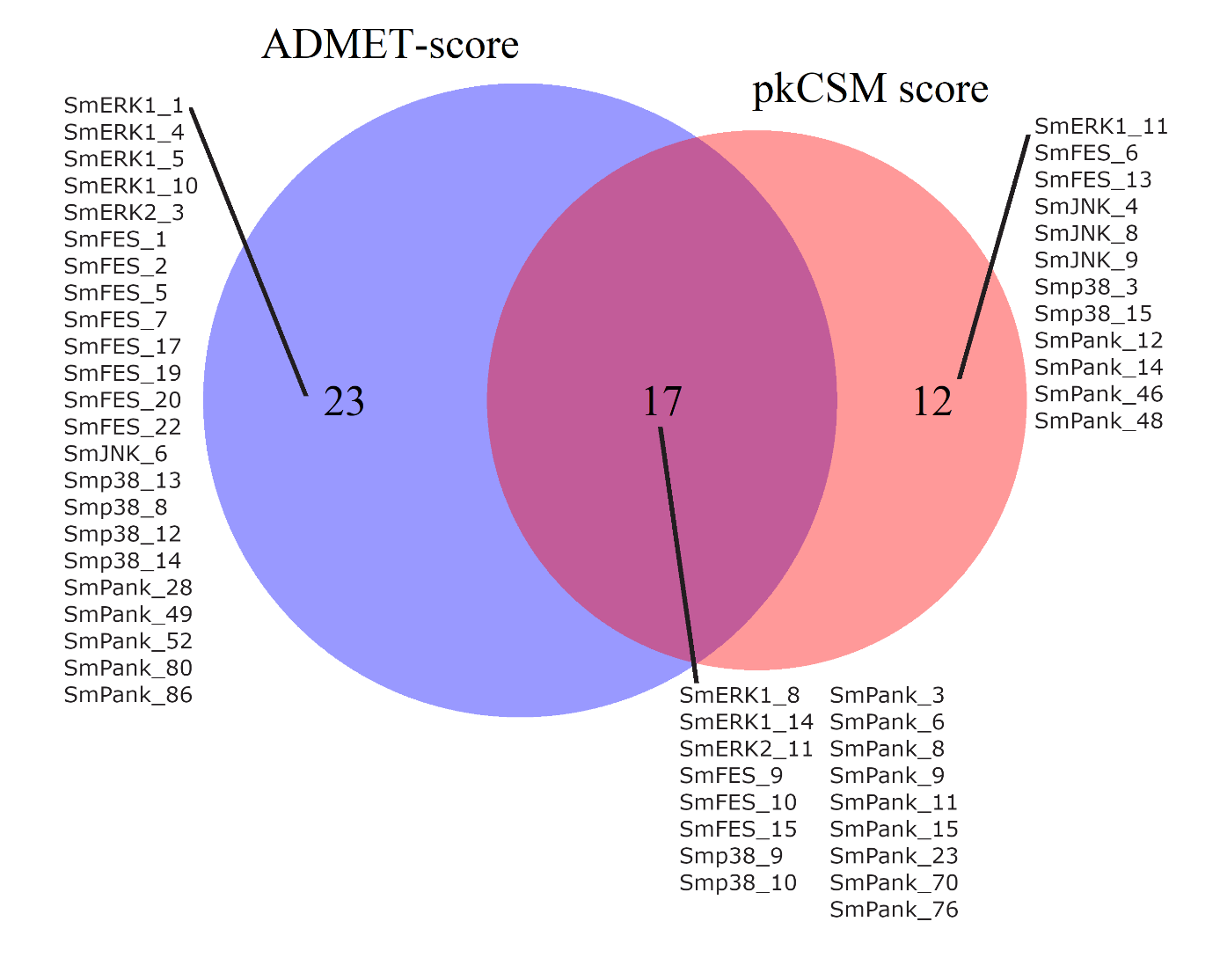


**Supplementary Figure S9: Druglikeness of active schistosomicidal compounds.** Venn diagram representing the distribution of the active compounds with pkCSM score (red) and/or ADMET-score (blue) greater than or equal to the score obtained for praziquantel.
